# Enzymatic mechanism of MlrB for catalyzing linearized microcystins by *Sphingopyxis* sp. USTB-05

**DOI:** 10.3389/fmicb.2024.1389235

**Published:** 2024-04-22

**Authors:** Junhui Teng, Qianqian Xu, Haiyang Zhang, Ruipeng Yu, Chao Liu, Meijie Song, Xiaoyu Cao, Xinyue Du, Suxuan Tao, Hai Yan

**Affiliations:** ^1^School of Chemistry and Biological Engineering, University of Science and Technology Beijing, Beijing, China; ^2^Beijing Institute for Drug Control, Beijing, China

**Keywords:** linearized microcystins, MlrB, biodegradation, active site, mechanism

## Abstract

Microcystins (MCs) are the most widespread cyanobacterial toxins in eutrophic water body. As high toxic intermediate metabolites, linearized MCs are further catalyzed by linearized microcystinase (MlrB) of *Sphingopyxis* sp. USTB-05. Here MlrB structure was studied by comprizing with a model representative of the penicillin-recognizing enzyme family via homology modeling. The key active sites of MlrB were predicted by molecular docking, and further verified by site-directed mutagenesis. A comprehensive enzymatic mechanism for linearized MCs biodegradation by MlrB was proposed: S77 transferred a proton to H307 to promote a nucleophilic attack on the peptide bond (Ala-Leu in MC-LR or Ala-Arg in MC-RR) of linearized MCs to form the amide intermediate. Then water was involved to break the peptide bond and produced the tetrapeptide as product. Meanwhile, four amino acid residues (K80, Y171, N173 and D245) acted synergistically to stabilize the substrate and intermediate transition states. This study firstly revealed the enzymatic mechanism of MlrB for biodegrading linearized MCs with both computer simulation and experimental verification.

## 1 Introduction

Microcystins (MCs) are the most common cyanotoxins produced by harmful cyanobacterial blooms and pose an increasing global threat to human health and ecosystems (Heresztyn and Nicholson, 1997; Neilan et al., 2013; Mariani et al., 2015). In mammals, MCs have been shown to be hepatocyte selective, liver tumor initiators and promoters, and irreversible inhibitors of serine/threonine protein phosphatases PP1 and PP2A (Ohta et al., 1994). Over 270 different MCs isomers have been discovered (Bouaïcha et al., 2019). Biodegradation is an effective and sustainable method for removing MCs. Both microbial communities and individual strains had shown the ability to degrade MCs (Bourne et al., 1996; Yan et al., 2012; Lezcano et al., 2016). However, in terms of potential environmental safety risks, the direct use of microorganisms in biotherapeutics can be problematic (Liu et al., 2020). Therefore, there has been a growing interest in the application of active enzymes for the MCs biodegradation.

In general, the peptides biodegradation pathway tends to follow a conversion that peptides to dipeptides and then to amino acids. Three enzymes have previously been shown to be critical in the *mlr-*dependent MCs biodegradation pathway (Bourne et al., 1996, 2001; Ishii et al., 2004; Shimizu et al., 2012). The first enzyme MlrA converts the structurally stable cyclic MCs into linearized MCs by opening the peptide bond between 3-amino-9-methoxy-2,6,8-trimethyl-10-phenyl-4,6-decadienoic acid (Adda) and arginine. The second enzyme MlrB further cleaves the peptide bond between alanine and leucine in the linearized peptide chain of MCs to produce the tetrapeptide compound. The third enzyme MlrC is responsible for the further degradation of tetrapeptide compounds to produce Adda. Presumably, adda is completely degraded from the phenylacetic acid degradation pathway to the tricarboxylic acid cycle (Zhang X. et al., 2020). Although the pathways for biodegrading MCs have been identified, the fundamental mechanisms of regulation of the biodegradation are not clear.

In order to further understand the mechanism of these three enzymes, an in-depth research for their active sites has become a hot topic. Heterologously expressed enzymes can be used not only to study their own functions and properties, but also to investigate biochemical pathways and molecular mechanisms (Zhang Z. et al., 2020). Mechanism of catalysis of enzyme A and enzyme C were studied in depth, and the crystal structure of enzyme C was analyzed to clarify its structure and function (Guo et al., 2023). However, information about MlrB is limited. The sequence of the MlrB enzyme is similar to members of the penicillin-recognizing enzyme family with the conserved sequence “Ser-Xaa-Xaa-Lys” (Xaa represents any amino acid, and Ser and Lys represent serine and lysine respectively), resulting in a preliminary classification as a serine hydrolase (Coque et al., 1993). This family comprises members such as β-lactamases (classes A, C, and D) and penicillin-binding proteins (Bourne et al., 2001; Shimizu et al., 2012). Phenylmethyl sulfonyl fluoride (PMSF) was found to effectively inhibit MlrB activity and further confirmed that it is a serine protease (Bourne et al., 1996). Dziga et al. (2016) validated the significance of the conserved sequence “Ser-Xaa-Xaa-Lys” in the activity center of MlrB by mutation experiments. By replacing Ser at position 77 (S77) and Lys at position 80 (K80) with other amino acids, they observed that the MlrB enzyme lost activity toward linearized MCs toxins. However, the specific binding sites between MlrB and linearized MCs and the biocatalytic mechanism have not yet been clearly clarified.

The optimal purification scheme for pure MlrB has been obtained in previous studies (Teng et al., 2023). Purified MlrB enzyme from *Sphingopyxis* sp. USTB-05 was successfully obtained using recombinant *Escherichia. coli* (*E. coli*) overexpression and affinity purification in previous studies (Xu et al., 2020; Teng et al., 2023). MlrB with low concentration was able to biodegrade linearized MCs within 30 min completely (Teng et al., 2023). The functions of enzymes are related to their structural features. However, the crystal structure of MlrB has not yet been resolved, and the structural details are poorly known, in particular its three-dimensional (3D) structure and the spatial distribution/location of the active site. Therefore, it is crucial to conduct 3D structural analyses of MlrB proteins.

In this study, the MlrB gene from *Sphingopyxis* sp. USTB-05 was successfully established heterologous expression of in *E. coli* and conducted site-directed mutagenesis to investigate the biodegradation mechanism of linearized microcysteinase on linearized MCs. Additionally, a homology model was used to predict the structure of linearized microcysteinase. By employing molecular docking technology, the binding modes between linearized microcysteinase and ligands such as linearized MC-LR and MC-RR were constructed to explorate the potential binding interactions. This work provides practical evidence for understanding the enzymatic mechanisms involved in MCs biodegradation by elucidating the role of bacterial linearized microcystinase in the toxic biodegradation of MC-LR and MC-RR.

## 2 Materials and methods

### 2.1 Physicochemical property analysis and homology modeling

Three major databases, AlphaFoldDB, UniProt and Swiss-Model, were searched for proteins with high similarity to MlrB from *Sphingopyxis* sp. USTB-05. Physicochemical properties of enzymes are available from the ExPAsy ProtParam online server. Signal peptide and transmembrane region structures were predicted using SignalP 5.0 and TMHMM 2.0, respectively. The Protein Structure Prediction Server (PSIPRED) and SOPMA were used for online secondary structure prediction. In the normal mode of Phyre2 website (Kelley et al., 2015), the full-length amino acid sequence was input for tertiary structure prediction and model construction, and the simulation results from the AlphaFold Protein Structure Database was compared. All software packages build the MlrB model based on the same template, and the final model evaluation is presented in a Ramachandran plot.

### 2.2 Molecular docking

The molecular structures of linearized MC-LR, linearized MC-RR and the docking ligands of linearized microcystinase were drawn using software. Autodock Vina was used to calculate the binding modes and the affinities of the linearized MCs to MlrB (Trott and Olson, 2010). The ligand was hydrogenated and the receptor protein was subjected to pre-processing such as adding polar hydrogen and calculating charge, and then the docking box was built (complete wrapping of the receptor protein). For each initial conformation, up to 9 generated binding conformations can be recorded. The docking results of the optimal binding model between receptor and ligand were visually displayed using Pymol 2.3 (DeLano, 2002).

### 2.3 Site-directed mutagenesis and expression

Based on our previous studies cloning and expressing MlrB from *Sphingopyxis* sp. USTB-05, derived from freshwater lake substrate, for linearized MC-RR biodegradation (Wang et al., 2015). Initially, the linearized microcystinase gene *mlrB* from *Sphingopyxis* sp. USTB-05 was amplified via polymerase chain reaction (PCR). In the second step, an overlap-extension PCR was used to construct the fixed point mutation of the *mlrB* gene. The oligonucleotide primers are listed in Table 1, and a detailed oligonucleotide primer scheme is provided by Sambrook and Russell (2016). The PCR products and the pET30a (+) vector plasmid were digested with *Bam*H I and *Xho* I restriction enzymes, respectively, and ligated with T4 DNA ligase. The constructed recombinant plasmid was transformed into *E. coli* DH5α cloning vector and positive clones were screened in antibiotic LB medium and sequenced by Sangon Biotech (Shanghai) Co., Ltd. The plasmid was extracted from *E. coli* DH5α/pET30a (+)/*mlrB* using the TIANprep mini plasmid kit (Tiangen, Beijing China) and transferred into the *E. coli* BL21 (DE3) expression vector, and positive clones were selected and further verified by gene sequencing. Consistent with previous work, the recombinant MlrB mutants (MlrB S77A, MlrB K80A, MlrB Y171A, MlrB N173A, MlrB D245A, MlrB H307A) were constructed and expressed successful (Wang et al., 2015). In the case of MlrB S77A, for example, the mutation is the replacement of the Ser at position 77 with an Ala, and so on. The bacteria cells were collected and washed three times using phosphate buffer solution (PBS, pH 7.4) and subsequently frozen in −20°C.

**Table 1:** List of primers on the forward, reverse, and mutant primers.

Primers	Primer Sequences (5′→3′)
*mlrB* F	ggatcc ATGACTGCAACAAAGCTTTTCCTGG
*mlrB* R	ctcgag CTACGGAAGCCGTCTGAACTCTAT
*mlrB* S77A Fm	CGAACTGGCG**GCA**ACATCGAAGCAGTTCACG
*mlrB* S77A Rm	GCTTCGATGT**TGC**CGCCAGTTCGAAGCGTG
*mlrB* K80A Fm	TCAACATCG**GCG**CAGTTCACGGCCGCTCTCA
*mlrB* K80A Rm	CGTGAACTG**CGC**CGATGTTGACGCCAGTTCG
*mlrB* Y171A Fm	CGTTTTTCC**GCC**GTCAACACCAATTACTTCC
*mlrB* Y171A Rm	GGTGTTGAC**GGC**GGAAAAACGACGGCCAGG
*mlrB* N173A Fm	CCTACGTC**GCC**ACCAATTACTTCCTGCTCG
*mlrB* N173A Rm	AATTGGT**GGC**GACGTAGGAAAAACGACGGCC
*mlrB* D245A Fm	GGCTATGGC**GCC**CGCGGCGTGCGGACTAATG
*mlrB* D245A Rm	CGCCGCG**GGC**GCCATAGCCTTGCCAGGTCC
*mlrB* H307A Fm	GTTGTGTCG**GCT**TCGGGCTTGGTTGTAGGC
*mlrB* H307A Rm	CCAAGCCCGA**AGC**CGACACAACACGCTCGC

*mlr*B F and *mlr*B R are forward and reverse primers, respectively. Fm and rm are mutant primers and the mutated sites introduced in the mutant primers are underlined.

### 2.4 Purification of mutant recombinants

Buffers were precooled to 4°C and all subsequent steps were performed on ice. Afterward, the cell pellet was resuspended in PBS (pH 7.4). Cells were disrupted by sonication and cell debris was removed by centrifugation (20 min, 22,351 ×g, 4°C). After filtration, the supernatant was mixed for 1 h with a Ni-NTA agar resin (Sangon Biotech, Shanghai, China). As a result, MlrB enzyme was firmly attached to the resin. The resin, which contained bound target proteins, was washed with a gradient of 5–10 column volumes of imidazole-containing PBS (pH 8), and the mobile fraction’s absorbance at 280 nm was monitored using Nanodrop (Thermo Fisher Scientific, Waltham, MA, USA) until it approached baseline level. Following that, the target proteins were isolated using PBS (125 mM imidazole, pH 8.0). The target proteins were ultimately removed imidazole and concentrated using ultrafiltration. It was necessary to assess the expression level of active MlrB by quantifying total soluble protein concentration according to Bradford (1976). Sodium dodecyl sulfate-polyacrylamide gel electrophoresis (SDS-PAGE) to check purified protein quality.

### 2.5 Determination of enzyme activity

The MlrA, prepared according to previous methods, was used to produce the first biodegradation products (linearized MCs) of 12.5 mg/L MC-LR and MC-RR (Xu et al., 2020). MC-LR and MC-RR were each mixed with 53.8 mg/L of MlrA and allowed to fully react at 30°C, 200 rpm for 3 h to produce linearized MCs with a final concentration of 12.5 mg/L. In order to verify the biodegradation activity of the recombinant MlrB enzyme toward the linearized products, two experimental groups and two control groups were formed by adding linearized MC-LR and MC-RR to solutions containing the MlrB enzyme (at a final concentration of approximately 1 mg/mL) and without the enzyme (control). Incubate the reaction system [30°C, 200 revolutions per minute (rpm)] and remove 200 μL of sample to a centrifuge tube at 0, 5, 15, 30, 45, and 60 min (min), then immediately add 2 μL of concentrated hydrochloric acid to stop the enzymatic reaction. All samples were centrifuged at 14,000 rpm for 20 min. Following the procedure described above, the activities of the six MlrB enzyme mutants were determined. Processed samples were stored at 4°C for subsequent monitoring of linearized MCs concentration by high-performance liquid chromatography (HPLC). The HPLC system (Shimadzu LC-20AT, Shimadzu Co., Ltd., Tokyo, Japan) was equipped with an ultraviolet Diode Array Detector at 238 nm using an Agilent TC-C18 column (4.6 mm × 250 mm; Agilent, 1200 series, Wilmington, DE, United States), in which the mobile phases for linearized MC-LR and MC-RR were 36% and 40% (v/v) acetonitrile-water solution containing 0.05% (v/v) trifluoroacetic acid, with a flow rate of 1.0 mL/min and an effective injection volume of 20 μL. All other reagents are chromatographic grade.

## 3 Results

### 3.1 Bioinformatics analysis of MlrB

To comprehensively elucidate the physicochemical properties of the MlrB (also known as USTB-05-B enzyme in *Sphingopyxis* sp. USTB-05) enzyme and make systematic structural predictions, a range of bioinformatic analysis software were used, including ProtParam, ProtScale, SignalP 5.0, TMHMM 2.0, PSIPRED, SOPMA, Phyre2, SWISS-MODEL, AlphaFoldDB and SAVES. ProtParam analysis revealed that the MlrB enzyme consists of 541 amino acid residues, of which alanine (11.5%) is the most abundant and cysteine (0.4%) the least abundant. There are 65 positively charged amino acid residues and 66 negatively charged amino acid residues. The theoretical isoelectric point (pI) of MlrB is 6.78, which is clearly different from most of the proteins in Table 2 and suggests that it is a biased acidic protein. The molecular formula is C_2635_H_4160_N_746_O_779_S_10_. The half-life in *E. coli* was more than 10 h, the *in vitro* instability coefficient was 24.85 (less than 40), and the aliphatic index was 89.48 (greater than 80), indicating that the protein was stable and beneficial for *in vitro* experiments (John Flensburg, 2005).

**Table 2:** Characterizing proteins related to MlrB.

ID	Organism	Length (AA)	Molecular weight (kD)	Theoretical isoelectric point	Instability index
M4NG93	*Sphingomonas* sp. USTB-05	541	59.08	6.78	24.85
A0A1W7MBK5	*Novosphingobium* sp. MD-1	541	59.09	8.77	23.57
A0A3B7DRB8	*Novosphingobium* sp. THN1	541	59.09	8.91	23.25
A0A141R8A3	*Sphingomonas* sp. ACM-3962	541	59.14	8.31	24.88
A0A4V1G4S8	*Sphingopyxis* sp. X20	541	59.14	8.3	25.81
A0A7X8NVI4	*Novosphingobium* sp. ERW19	541	59.09	8.91	23.25
D0FYG3	*Sphingopyxis* sp. C-1	541	59.06	8.91	25.39
A0A1D8QSQ4	*Sphingopyxis* sp. MB-E	537	58.55	6.35	23.53
A0A1X9PWL1	*Sphingopyxis* sp.	528	57.71	6.54	25.43
A0A7X8S5T1	*Novosphingobium* sp. ERN07	526	57.55	8.78	24.04
SMTL ID: 1ei5.1	*Ochrobactrum anthropi*	529	57.39	5.12	38.57

The amino acid sequence of USTB-05-B was analyzed using SignalP 5.0 signal peptide software. There is a signal peptide sequence of MlrB, which consists of amino acids at positions 1 to 19, with a break point between serine at position 19 and histidine at position 20 of the sequence, and then the amino acids at positions 20 to 541 to form a mature chain. The TMHMM2.0 server predicted no transmembrane sequence in MlrB, and MlrB was assumed to be a secreted protein. The hydrophilicity of MlrB was predicted with ProtScale software. The number of hydrophilic amino acid residues in MlrB’s polypeptide chain was significantly higher than that of hydrophobic residues, suggesting MlrB is a hydrophilic protein. Based on SOMPA prediction analysis, MlrB contains four secondary structures, alpha helix (30.87%), random coil (42.88%), extended strand (19.04%), beta turn (7.21%), indicating that the major component of the secondary structure is the random coil, followed by the alpha helix. PROSITE analysis indicates that MlrB belongs to the class A beta-lactamases and possesses one active site situated at amino acid positions 73–88.

**Figure 1:**
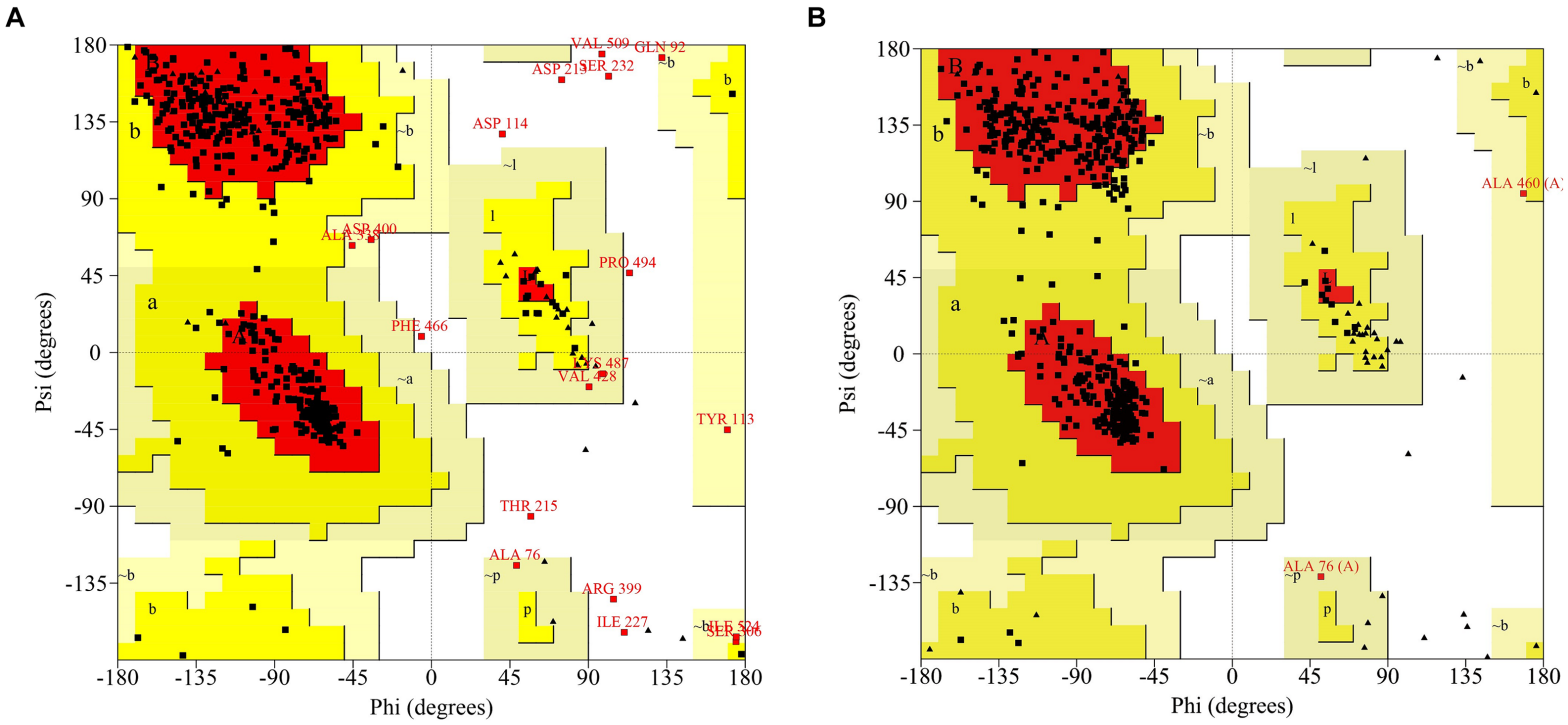
Ramachandran Plot to assess models of MlrB. **(A)** The model constructed by phyre2. **(B)** The model constructed by Alphafold (accessions: M4NG93).

Phyre2 was used for the MlrB similarity search and the coverage of the first high score template with MlrB was 96%. MlrB shows 100% homology with the template and 91% of protein residues are modeled with 100% confidence. The highest-scoring template (Protein Data Bank database ID: 1ei5; chain A) used for modeling was the D-aminopeptidase (DAP) from *Brucella anthropi*, which belongs to the new member of the “penicillin-recognizing enzyme” family (Bompard-Gilles et al., 2000). The sequence identity of the template to MlrB is 25%, which is below the lower limit of expected accuracy (typically 30%; Fiser, 2010), but the high confidence level observed (>91%) suggests that the target MlrB sequence may be a true homolog of the template, and therefore ensures the reliability of the modeling. The model was evaluated using the Ramachandran plot, revealing that 88.1% (378/429) of amino acid residues were located in the most favored regions and 98.1% (421/429) of amino acid sites were located in permissive regions in the model constructed by phyre2 (Figure 1A). Correspondingly, alphafold model (accessions:M4NG93) shows that 89.6% (412/460) of the amino acid residues are in the most favorable region, with the percentage increases to 100% in the permissive region (Figure 1B). The results demonstrate that the model constructed by the latter is more accurate and reliable, making it suitable for further docking analyses.

**Figure 2:**
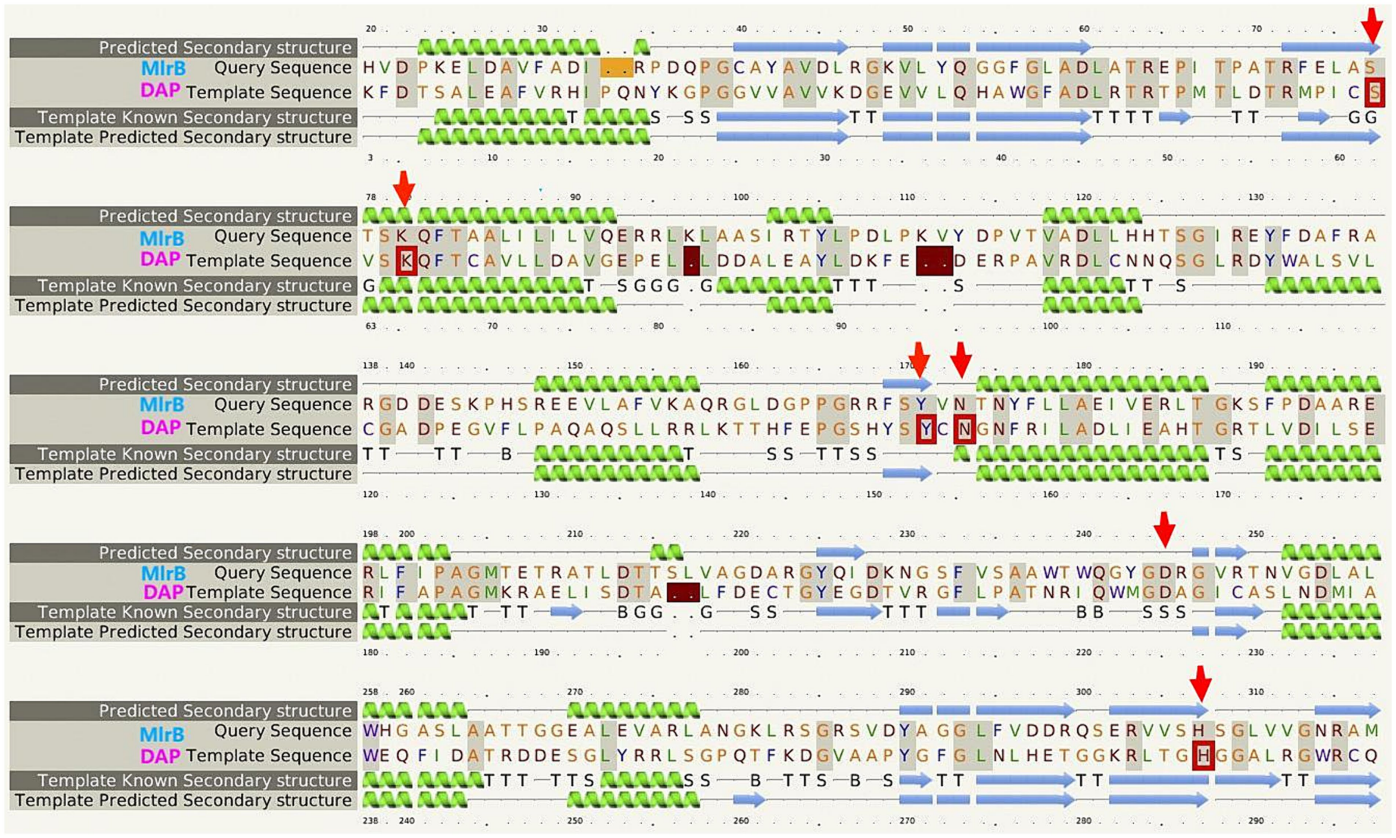
Multiple sequence alignment of the template enzyme DAP (PDB ID: 1ei5; chain A) and the MlrB (AGG86526.1 from *Sphingopyxis* sp. USTB-05).

Multiple sequence comparison of the template enzyme DAP (1ei5, chain A) with MlrB (AGG86526.1) is shown in Figure 2. The alpha helix is represented by the green helix, the β-folding by the blue arrow and the conserved active sites of DAP by the five red boxes. The six red arrows point to possible key sites for MlrB, serine at position 77 (S77), lysine at position 80 (K80), tyrosine at position 171 (Y171), asparagine at position 173 (N173), aspartic acid at position 245 (D245) and histidine at position 307 (H307).

### 3.2 The substrate-binding mode of MlrB

**Figure 3:**
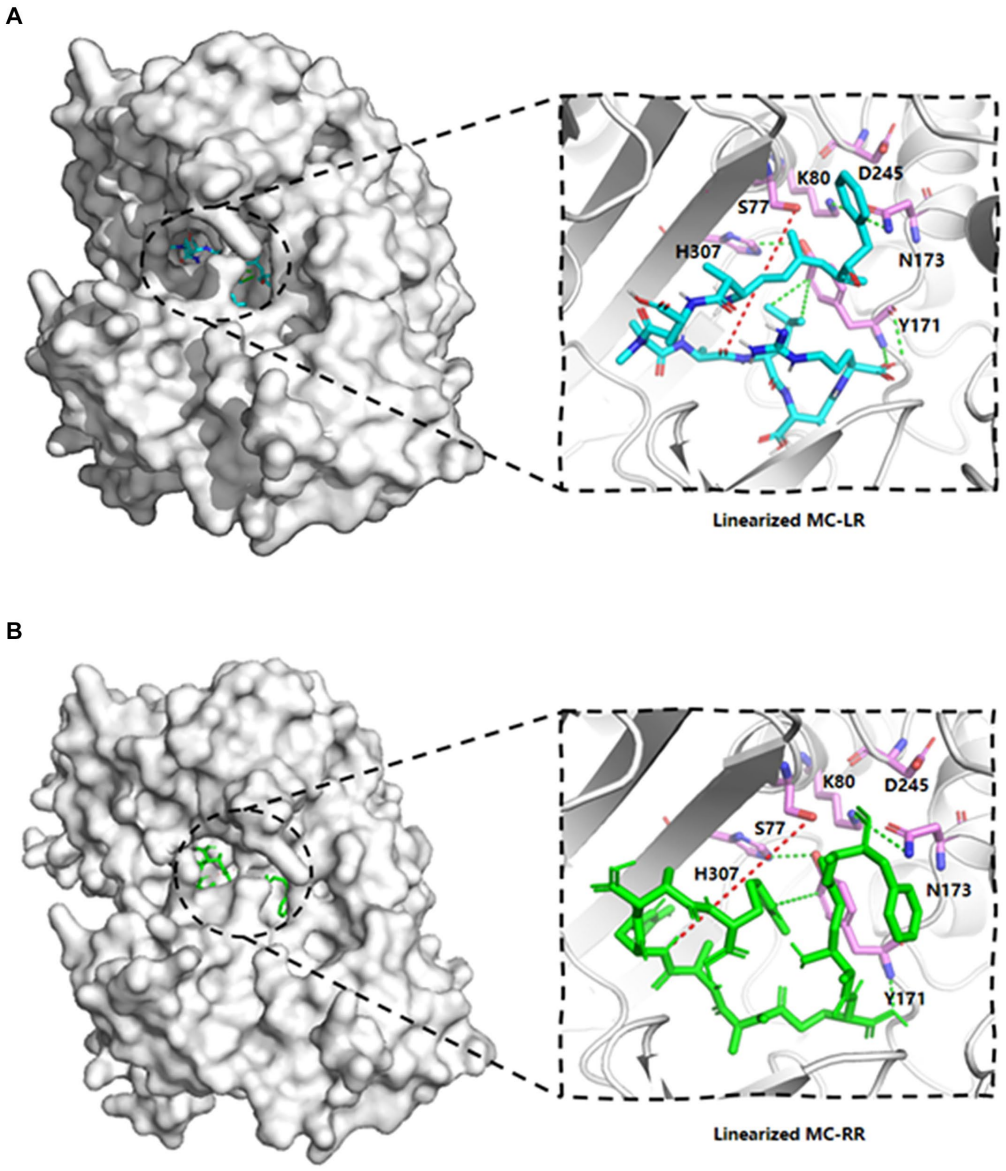
Linearized MC-LR and MC-RR molecules docked to cartoon depiction of MlrB. **(A)** Linearized MC-LR molecules docked to MlrB. **(B)** Linearized MC-RR molecules docked to MlrB. The linearized MC-LR and MC-RR are colored blue and green respectively, while the receptor proteins are all colored gray, with the positions of the six active sites shown as stick form. Colored cyan is the receptor-ligand interaction force, while the site where the receptor hydrolyses the ligand is denoted by a red dotted line. All active sites of MlrB are surrounded by the structural center, and the spatial arrangement of the six active sites is apparent.

The template’s high homology and coverage allow us to speculate about the six potential key sites of MlrB (S77, K80, Y171, N173, D245 and H307) based on DAP’s mechanism of action. The results of molecular docking further substantiated our hypothesis regarding the enzyme catalytic process through computer simulation. The docking scores of the linearized microcystinase model with linearized MC-LR and MC-RR were -8.8 kcal/mol and -8.3 kcal/mol, respectively, and the binding energies were much less than -5 kcal/mol, indicating that the enzyme binds to the substrate with high affinity (Ge et al., 2023). As depicted in Figure 3, the substrate molecules were fully accommodated within the active pocket. Interestingly, the spatial positions of the six active sites in MlrB enzyme coincide with the positions of the catalytically active residues in the template enzyme DAP. It was evident that Y171 occupied an optimal position with respect to the substrate, while the other five amino acids surrounded the substrate molecules. D245 is positioned close to the N terminus of the substrate, whereas S77, K80 and H307 are situated on one side of the substrate, with Y171 and N173 located on the opposite side, thereby encapsulating the substrate within. Analysis of receptor-ligand interactions revealed multiple direct hydrogen bonds between Y181 and the substrate molecules, which could potentially be crucial for catalysis. However, it should be noted that molecular docking is a computational method that often lacks receptor flexibility and thus introduces uncertainty into protein-ligand complex reliability (Bompard-Gilles et al., 2000). Therefore, it is necessary to supplement experimental evidence to ensure the theoretical conjecture. Using genetic engineering techniques, mutant enzymes can be constructed, purified and subsequently mixed with linearized MCs to verify each site’s role in degradation experiments, thereby revealing the mechanism of linearized MCs biodegradation by enzyme MlrB.

### 3.3 Purification of mutant recombinants MlrB

**Figure 4:**
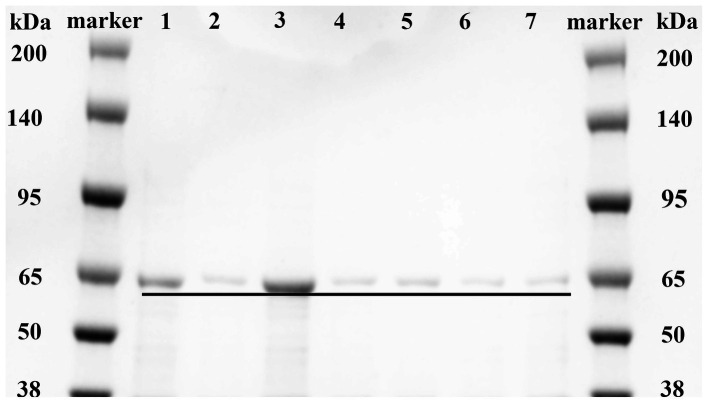
Purification of recombinant MlrB and its site-directed mutants. Lane 1 represents the MlrB enzyme without the mutation (MlrB). Lane 2 represents the MlrB enzyme with a mutation at position 77, substituting serine with alanine (MlrB S77A). Lane 3 represents the MlrB enzyme with a mutation at position 80, substituting lysine with alanine (MlrB K80A). Lane 4 contains the MlrB enzyme responsible for converting tyrosine at position 171 into alanine (MlrB Y171A). Lane 5 contains the MlrB enzyme responsible for converting asparagine at position 173 into alanine (MlrB N173A). Lane 6 represents the MlrB enzyme that changes aspartic acid to alanine at position 245 (MlrB D245A). Lane 7 corresponds to the MlrB enzyme that alters histidine to alanine at position 307 (MlrB H307A).

Based on the analysis of the molecular docking binding pattern results, six mutants of MlrB were constructed using previous method (Teng et al., 2023). After inducing the expression of the target protein, we conducted chromatography purification using affinity, concentrated the sample by ultrafiltration, and analyzed by SDS-PAGE. Successful purification of the six mutants and a positive control was confirmed, as shown in Figure 4. Lane 1 represents the control group (wild-type MlrB), while lanes 2 to 7 represent the mutant groups. The molecular weight of the MlrB is theoretically 59 kDa. It should be noted that the expected band, ranging from 50 kDa to 65 kDa, has been obtained and underlined, MlrB and its mutations were purified successfully.

### 3.4 Enzyme activity

**Figure 5:**
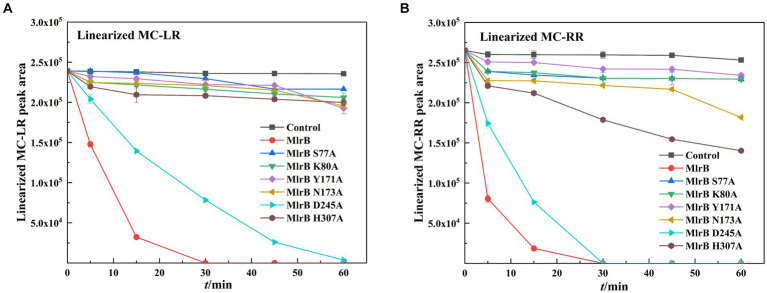
Biodegradation kinetics of linearized MC-LR and MC-RR by MlrB and mutants. **(A)** Linearized MC-LR **(B)** Linearized MC-RR.

The biodegradation kinetics of MCs shown for linearized MC-LR, point mutants of S77A, K80A, Y171A, N173A and H307A resulted in a complete loss of enzyme activity (Figure 5A). The D245A mutation greatly reduces MlrB activity, requiring 1 h to fully biodegrade linearized MC-LR. Interestingly, aspartate also is not a major site for DAP (Bompard-Gilles et al., 2000). For linearized MC-RR, S77A, K80A, Y171A, N173A, H307A point mutants also resulted in complete loss of enzyme activity (Figure 5B). D245A mutations significantly reduced MlrB activity.

## 4 Discussion

In this study, the technique of molecular docking emerged as a powerful tool for exploring the molecular mechanisms of protein-ligand and protein–protein interactions, a technique commonly used to explore the possible binding modes of substrates to their bioactive molecules (Pingaew et al., 2018; Zulkipli et al., 2020). Obtaining a high-quality structure of MlrB is crucial. Therefore, the protein models predicted by two software programs were evaluated using the Ramachandran plot. Molecular docking simulations indicated that certain conserved amino acid residues of the MlrB formed catalytic pockets to which the linearized MCs are held intact by hydrogen bonding, van der Waals forces, hydrophobic and electrostatic interactions, etc. Amino acid residues in the active site are involved in breaking linearized MCs into tetrapeptides. Although the tetrapeptide is still toxic, it acts as an indispensable stage in the detoxification process of linearized MCs. Based on the results of molecular docking and enzymatic degradation experiments, the mechanism of linearized MCs biodegradation by MlrB was speculated. MlrB is classified as a typical serine protease. By comparing its active site with that of the optimal template DAP enzyme (1ei5; chain A), there was a found that MlrB also contained three conserved motifs of serine penicillin-recognizing enzymes (Bompard-Gilles et al., 2000). These are mainly involved in six amino acids (S77, K80, Y171, N173, D245, H307). These non-adjacent amino acids form a catalytic pocket during enzyme-substrate binding. The pocket contains the “oxygen anion cavity” (His307 and Asn173) that stabilizes the tetrahedral intermediate and the general alkali that extracts the proton from the serine hydroxyl group. The substrate is surrounded by the pocket in which the amino acid residues participate either directly or indirectly in the recognition and catalysis process.

**Figure 6:**
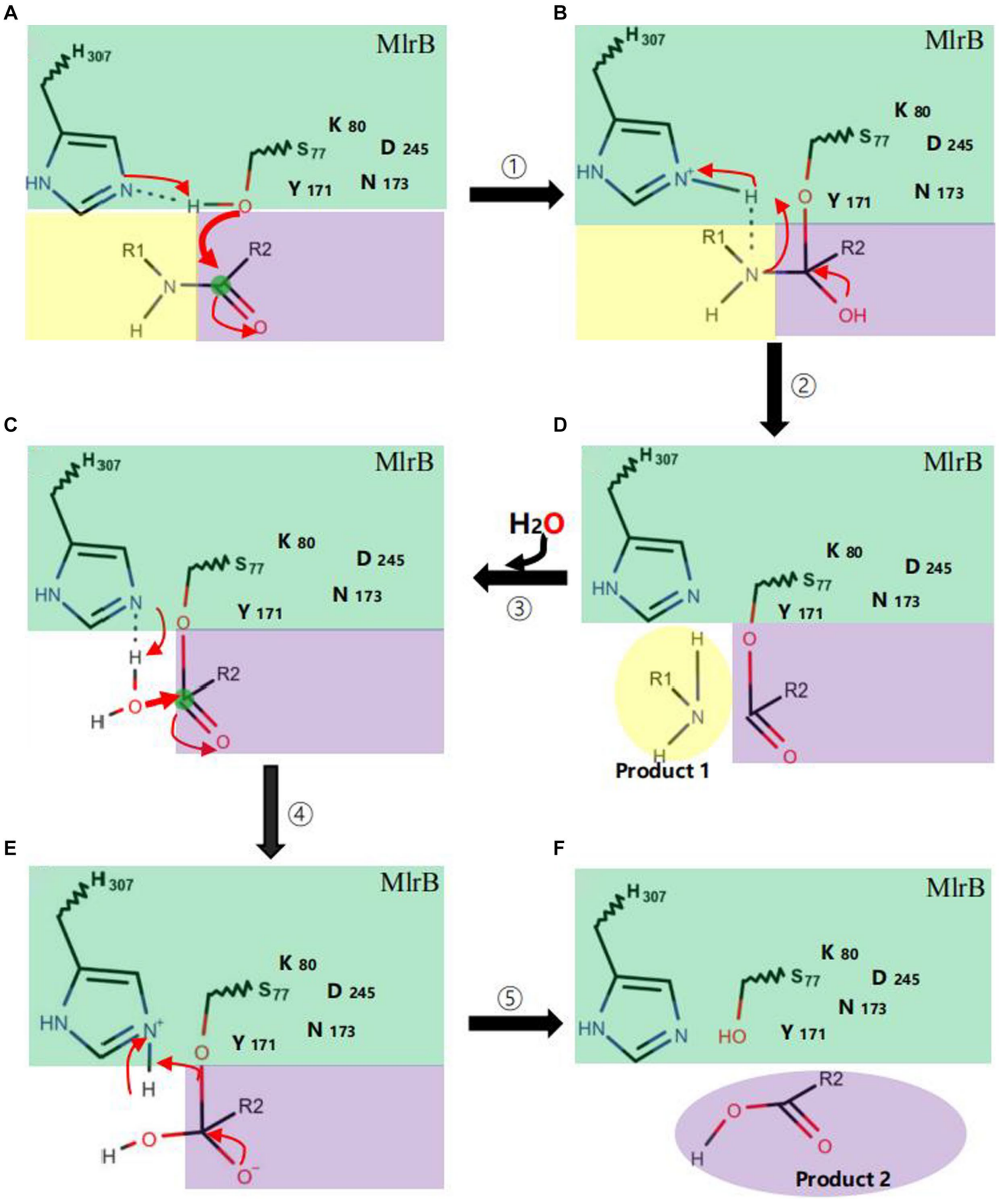
Catalytic mechanism of MlrB enzyme. Acylation reactions (① **A**→**B** and ② **B**→**D**), deacylation reactions (③–⑤ **D**→**C**→**E**→**F**). The MlrB enzyme is represented in green, while the yellow and purple colors represent the linearized MCs. The path of electron transfer and nucleophilic attack is indicated by red arrows.

The mechanism could be described as follows (Figure 6): linearized MCs bind to MlrB, and the peptide bond is then wrapped by the enzyme’s active sites. Firstly, in the acylation reaction (Figure 6, ① and ②), the nitrogen on H307 extracts a proton from the hydroxyl group on S77. The hydroxyl group then acts as a nucleophilic reagent and attacks the carbonyl carbon of the peptide bond (Ala-Leu in MC-LR or Ala-Arg in MC-RR) of linearized MCs. The oxygen in the carbonyl group of the peptide bond receives a pair of electrons from the double bond, creating a tetrahedral intermediate. The bond between nitrogen and carbon is broken. The covalent electrons that formed the bond then attack the hydrogen of H307, breaking the connection between nitrogen and hydrogen and releasing the first product. The electron previously transferred to the oxygen moves back to reform the double bond, producing a covalent acyl-enzyme complex. Subsequently, water is added to the deacylation reaction (Figure 6, ③–⑤), the nitrogen of H307 accepts a proton from the water, which replaces the N-terminus of the cleaved peptide and attacks the carbonyl carbon. When a bond is formed between the oxygen of the water and the carbonyl carbon, the electrons in the double bond move to the oxygen, making it negative and creating a second tetrahedral intermediate. The bond between S77 and the carbonyl carbon which formed in the first step attacks the hydrogen of H307. Finally, the hydrogen transfers back to S77. The transition state disintegrates and the second product, the tetrapeptide, is released. Dziga et al. (2016) reported S77 as an important site in the degradation of linearized MCs to tetrapeptides, which is consistent with our findings. We suggested that Y171 and N173 placed H307 in a favorable position for hydrogen extraction. The Nδ2 atoms of Y171 and N173 form hydrogen bonds with K80, which reduces the ambient pKa to keep it unprotonated under neutral conditions and create the environmental conditions for proton transfer in the acylation and deacylation reactions. D245 and N173 may also be involved in the specific recognition of the N-terminal and C-terminal substrate, respectively. The formation of a hydrogen bond between the carboxyl group of D245 and H307 could further increase the electronegativity of the nitrogen of H307 and be involved in the stabilization of the amide intermediate to coordinate the reaction.

## Data availability statement

The datasets presented in this study can be found in online repositories. The names of the repository/repositories and accession number(s) can be found in the article/supplementary material.

## Author contributions

JT: Data curation, Methodology, Validation, Visualization, Writing – original draft, Writing – review & editing. QX: Conceptualization, Methodology, Project administration, Writing – review & editing. HZ: Formal analysis, Software, Validation, Writing – review & editing. RY: Data curation, Software, Validation, Writing – review & editing. CL: Writing – review & editing. MS: Writing – review & editing. XC: Writing – review & editing. XD: Writing – review & editing. ST: Writing – review & editing. HY: Funding acquisition, Resources, Writing–review & editing, Supervision, Project administration.
